# Ultrasound-derived diaphragmatic kinematic and morphometry parameters in children with cerebral palsy: a comparative cross-sectional study

**DOI:** 10.1007/s00431-026-06946-y

**Published:** 2026-04-22

**Authors:** Paulo A. F. Magalhães, Thálita R. L. Crispim, Fabianne M. N. A. Dantas, Helga C. Muniz, Bárbara Bernardo R. S. Figueiredo, Emanuelle F. D. Schmit, Cyda M. A. Reinaux

**Affiliations:** 1 Graduate Program in Rehabilitation and Functional Performance (PPGRDF), Universidade de Pernambuco, Petrolina, Pernambuco Brazil; 2 Research Group in Neonatology and Pediatrics (Baby GrUPE), Universidade de Pernambuco, Petrolina, Pernambuco Brazil; 3 Universidade Federal de Pernambuco (UFPE), Recife, Pernambuco Brazil

**Keywords:** Cerebral palsy, Diaphragm ultrasound, Respiratory muscle strength, Pediatrics, Respiratory physiotherapy

## Abstract

The purpose was. to evaluate diaphragmatic mechanics and morphometry using ultrasound in children with cerebral palsy (CP) compared to typically developing controls. By quantifying parameters such as excursion, contraction/relaxation velocity, and thickness, we aimed to distinguish primary respiratory muscle impairment from anthropometric and clinical confounders to inform targeted management. This comparative cross-sectional study included 102 participants: 51 children with spastic CP (GMFCS levels I–V) and 51 age- and sex-matched healthy controls. Diaphragmatic ultrasound was used to assess excursion, thickness and contraction/relaxation velocity. Multivariate linear regression models, adjusted for age, sex, and weight, as well as clinical confounders (medication use, prior surgeries, and history of pulmonary disease), were applied to determine independent associations with CP. In the unadjusted analysis, children with CP showed significantly reduced expiratory excursion (*p* = 0.00) and shorter respiratory times (*p* = 0.00) than controls, together with greater inspiratory thickness (*p* = 0.03) and contraction velocity (*p* = 0.01). After adjustment for demographic, anthropometric, and clinical confounders, the inspiratory differences were no longer significant, indicating that they reflected body size–related effects. In contrast, reduced expiratory excursion (*p* = 0.03) and shorter expiratory time (*p* = 0.01) remained independently associated with CP, particularly in nonambulatory participants (GMFCS IV–V).

*Conclusion*: Most observed differences in diaphragmatic mechanics and morphometry in children with CP are attributable to lower weight and related confounders. However, the persistent reduction in expiratory excursion and expiratory time, independent of anthropometric and clinical confounders, identifies the expiratory phase as a critical and distinct marker of respiratory impairment in children with CP. Clinical interpretation of diaphragmatic ultrasound in this population requires rigorous adjustment to avoid diagnostic misinterpretation.

**Table:** 

**What is Known:** • *Respiratory dysfunction is a major contributor to morbidity in children with cerebral palsy (CP), driven by neuromuscular impairment and altered chest wall mechanics.* • *Diaphragm ultrasound is a reliable, noninvasive tool for assessing respiratory muscle function, although its application in children with CP remains limited.*
**What is New:** • *After adjustment for demographic, anthropometric, and clinical confounders, most diaphragmatic parameters were not independently associated with CP.* • *Expiratory dysfunction (characterized by reduced expiratory excursion and shortened expiratory time) emerged as a distinct feature, suggesting that impaired abdominal muscle function may represent a previously underrecognized mechanism of respiratory dysfunction in children with CP.*

**Supplementary Information:**

The online version contains supplementary material available at 10.1007/s00431-026-06946-y.

## Introduction

Respiratory disease is the leading cause of premature mortality and reduced quality of life in children with cerebral palsy (CP) [1–3]. While the primary deficits in CP are neuromotor, the respiratory pump is invariably affected by the same pathophysiology: trunk muscle spasticity, poor postural control, and chest wall deformities [4–6]. These mechanical constraints compromise cough efficiency and ventilation, predisposing these children to recurrent lower respiratory tract infections and hospital admissions [6–9].

Central to this respiratory vulnerability is the diaphragm. As the primary muscle of inspiration, its function is often impaired by uncoordinated activation and chest wall restriction [10]. Preserving diaphragmatic mobility and strength is therefore critical for maintaining functional respiratory reserves [11, 12]. However, assessing respiratory muscle function in this population remains a significant clinical challenge. Conventional pulmonary function tests, such as spirometry, depend heavily on cognitive comprehension and volitional effort, often excluding children with severe motor or intellectual impairments from accurate evaluation.

To bridge this diagnostic gap, diaphragm ultrasound (DU) has emerged as a promising tool. It provides a noninvasive, radiation-free, and bedside method to quantify diaphragmatic excursion, thickness, and contractility [13]. Crucially, DU allows for the assessment of diaphragmatic function during quiet breathing, independent of the patient’s ability to perform maximal maneuvers [13]. Despite its established utility in other pediatric conditions, data characterizing specific diaphragmatic morphofunctional alterations in children with CP remain scarce.

Therefore, this study aimed to evaluate diaphragmatic function using ultrasound in children with CP compared to typically developing controls. By objectively quantifying parameters such as excursion, contraction and relaxation velocities, and thickness, we sought to elucidate the specific patterns of respiratory muscle impairment in this population, providing a physiological basis for more targeted respiratory management.

## Material and methods

### Study design and setting

This observational, cross-sectional study followed a matched case–control design and adheres to the STROBE Statement guidelines [14]. The protocol was approved by the Research Ethics Committee of the Centro Integrado de Saúde Amaury de Medeiros (CISAM/UPE; Protocol No. 60569722.6.0000.5191) and complied with the Declaration of Helsinki [15] and Brazilian National Health Council Resolution 466/2012. Written informed consent was obtained from parents or legal guardians prior to enrollment.

Data collection was conducted at the Cardiopulmonary Physiotherapy Laboratory (LAFIC) of the University of Pernambuco, serving as the centralized evaluation site. The study covered two municipalities in the Vale do São Francisco region, northeastern Brazil, and was conducted in partnership with regional neurorehabilitation centers.

### Participants and setting

This study was designed as a population-based investigation covering two municipalities in the Vale do São Francisco region, northeastern of Brazil. To ensure comprehensive identification of the target population, recruitment was conducted in strategic partnership with regional neurorehabilitation referral centers and public transport services exclusively dedicated to individuals with severe mobility impairments. All data collection was centrally performed at the LAFIC of the University of Pernambuco, serving as the sole standardized evaluation site to minimize measurement variability.

The study population comprised children aged 2 to 12 years, stratified into two groups. The cerebral palsy (CP) group consisted of children with a clinical diagnosis of CP confirmed by a pediatric neurologist. Exclusion criteria for this group included hemodynamic instability, history of thoracic surgery, presence of active chest drains, or lack of parental consent. The healthy control (HC) group was recruited from local schools using frequency-based matching for age and sex relative to the CP cohort. However, after eligibility screening and data quality procedures, a residual imbalance in sex distribution remained. To address this issue and ensure internal validity, sex was included as a covariate in all multivariable models. Controls were excluded if they presented with any respiratory illness within the previous six months, known cardiac or neurological conditions, obesity (BMI-for-age > 95th percentile), or evidence of growth failure.

Regarding therapeutic history, information on ongoing respiratory physiotherapy was recorded. To strictly control for acute physiological modifiers of diaphragmatic mechanics, participants were not evaluated immediately following rehabilitation sessions. Furthermore, no standardized airway clearance or respiratory training intervention was delivered as part of the study protocol; thus, physiotherapy history was treated as a chronic characteristic rather than an acute confounding variable.

### Sample size calculation

The sample size was determined a priori using G*Power software (v3.1.9.4) based on data from a pilot study involving 12 children with CP and 12 healthy controls. The calculation was based on the primary outcome requiring the largest sample size: inspiratory diaphragmatic thickness (pilot means: CP = 1.34 ± 0.28 mm vs. HC = 1.20 ± 0.16 mm). Assuming a standardized effect size of *d* = 0.61, an alpha error of 0.05, a power of 0.80, and an allocation ratio of 1:1, a minimum of 86 participants (43 per group) was required. To account for potential screening failures and technical limitations in ultrasound image acquisition, the target recruitment was increased to 51 participants per group.

### Data collection protocol

Each participant underwent a comprehensive, individualized evaluation conducted by a single trained physiotherapist, adhering to a standardized four-step sequence designed to ensure data reliability and minimize participant fatigue:

1. Anamnesis and anthropometry

Body mass (kg) was measured using a calibrated digital scale with 0.1-kg resolution, with the child barefoot and wearing light clothing, and was included as a continuous covariate to account for body size–related effects. Height was assessed according to functional status. Ambulatory participants were measured using standard standing stadiometry (0.1-cm resolution). In nonambulatory children or those with significant postural deformities (conditions that limit the accuracy and comparability of standing height), stature was estimated from knee height using a sliding caliper and converted using validated equations for children with cerebral palsy [16]. Arm span was not used, as upper-limb asymmetries and contractures are common in CP and may reduce measurement reliability. Owing to this methodological heterogeneity and its multicollinearity with age and weight, height was not retained in the multivariable models, and weight was selected as the primary anthropometric covariate.

(2) Clinical respiratory assessment

This step followed established pediatric semiology guidelines. The evaluation was performed with the child in a resting state, comfortably positioned to avoid anxiety-induced tachypnea.

- Thoracic inspection: Chest wall configuration was inspected for symmetry and structural deformities (e.g., pectus excavatum/carinatum and scoliosis). Chest type was dichotomized as *normal* or *altered*.
- Ventilatory pattern: Defined by the predominant regional displacement during quiet tidal breathing, categorized as *diaphragmatic* (abdominal predominance), *thoracic* (upper rib cage predominance), or *thoracoabdominal* (mixed).
- Respiratory amplitude: Qualitatively assessed by visual inspection of chest wall expansion and categorized as *preserved* or *reduced*.
- Pulmonary auscultation: Performed systematically across anterior, lateral, and posterior lung fields during quiet breathing. Findings were classified *as normal* (vesicular breath sounds present without adventitious sounds) or *altered* (presence of crackles, wheezes, or diminished breath sounds), serving as a proxy for current respiratory health status.

(3) Functional classification

Gross motor function was stratified using the Gross Motor Function Classification System (GMFCS)–Expanded and Revised [17]. Classification was determined through a combination of direct observation of the child’s mobility in the assessment environment and a structured interview with caregivers regarding performance in daily settings (home/school).

(4) Diaphragmatic ultrasound (DU)

The final step comprised the ultrasonographic assessment of diaphragm morphometry and kinematics, performed after a rest period to ensure baseline respiratory conditions.

All data were recorded prospectively on standardized case report forms. To ensure data integrity, entries were independently verified for completeness immediately post-assessment.

### Ultrasound assessment

Diaphragmatic ultrasound (DU) was performed with participants in a semirecumbent position (45° inclination) to optimize patient comfort and minimize abdominal muscle recruitment. Assessments were conducted strictly during quiet tidal breathing (resting breathing). This approach was chosen to standardize measurements across the sample, ensuring data validity regardless of the child’s cognitive ability or capacity to perform volitional respiratory maneuvers (e.g., maximal inspiratory efforts).

Diaphragm ultrasound was performed exclusively on the right hemidiaphragm to maximize image quality through the hepatic acoustic window. No measurements were obtained from the left side, and therefore, no averaging across hemidiaphragms was required. Although children with CP may present asymmetric trunk mechanics, right-sided acquisition was prioritized for standardization and reproducibility [13].

All ultrasound images were acquired by a single experienced physiotherapist to ensure consistency in transducer handling and positioning. Subsequently, to minimize detection bias, a second expert evaluator, completely blinded to the participants’ clinical data and group allocation, performed the offline measurements using stored video loops. To verify the reproducibility of this protocol, intra-rater and inter-rater reliability were assessed using the intraclass correlation coefficient (ICC), yielding values > 0.90 for all primary outcomes, indicating excellent reliability.

#### Morphometric assessment of diaphragm thickness

**Fig. 1:**
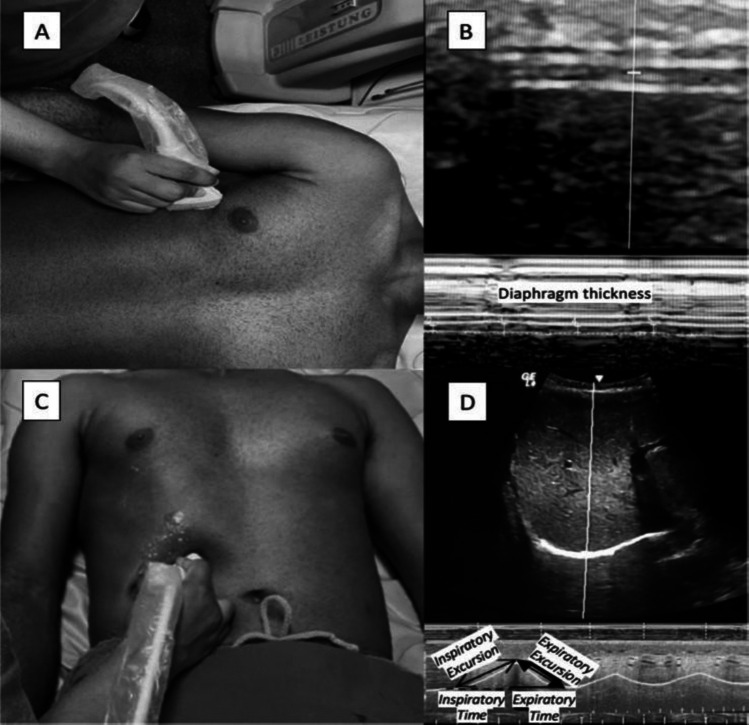
Diaphragmatic ultrasound assessment. (**A**) Probe positioning for acquisition of diaphragmatic thickness measurements. (**B**) Ultrasound image showing diaphragmatic thickness. (**C**) Probe positioning for functional diaphragmatic assessment. (**D**) Ultrasound image illustrating functional parameters: inspiratory excursion, expiratory excursion, inspiratory time, and expiratory time, 2025

To evaluate diaphragmatic thickness, a high-frequency linear transducer (7.5 MHz) was positioned at the right zone of apposition (typically between the 8th and 10th intercostal spaces, mid-axillary line) (Fig. 1A). The transducer was angled perpendicular to the chest wall to visualize the diaphragm as a characteristic three-layered structure: a central hypoechoic muscle layer bordered by two hyperechoic lines (the pleural and peritoneal membranes) (Fig. 1B) [18]. Diaphragmatic thickness (Tdi) was measured perpendicularly to the muscle fibers at two distinct phases:

- Inspiratory thickness (Tdi_insp): Measured at the peak of tidal inspiration, representing the contracted state.
- Expiratory thickness (Tdi_exp): Measured at the end of quiet expiration, representing the relaxed state. The final value for each parameter was derived from the mean of three measurements obtained from distinct respiratory cycles.

#### Diaphragm excursion

Diaphragm excursion was assessed using a 3.5-MHz convex transducer positioned immediately below the right costal margin along the mid-clavicular line, employing a subcostal trans-hepatic approach (Fig. 1C). The ultrasound beam was oriented medially, cranially, and dorsally to obtain a perpendicular view of the posterior third of the right hemidiaphragm. Recordings were performed during quiet tidal breathing, and five consecutive respiratory cycles were captured in M-mode for each participant. To minimize breath-to-breath variability and ensure measurement stability, the final values for excursion, thickness, and all kinematic variables were computed as the mean of three consecutive high-quality cycles, selected based on optimal dome visualization and trace reproducibility. From these standardized M-mode tracings, the diaphragmatic excursion parameters were subsequently derived:

- Diaphragmatic excursion (DE): The vertical amplitude (mm) of movement during the inspiratory (DE_insp) and expiratory (DE_exp) phases (Fig. 1D).
- Respiratory times: The duration of the inspiratory (T_insp) and expiratory (T_exp) phases, measured in seconds.
- Diaphragmatic velocities: Calculated to quantify the speed of muscle shortening and lengthening dynamics:
  - ⚬ Contraction velocity (mm/s): Defined as the ratio of inspiratory excursion to inspiratory time (DE_insp/T_insp).
  - ⚬ Relaxation velocity (mm/s): Defined as the ratio of expiratory excursion to expiratory time (DE_exp/T_exp).

These kinematic variables collectively provide a comprehensive profile of the diaphragm’s pump function, capturing both the magnitude of displacement and the velocity of fiber recruitment and recoil.

### Statistical analysis

Statistical analyses were performed using the Statistical Package for the Social Sciences (SPSS version 20.0; IBM Corp., Armonk, NY), following established guidelines for biomedical research [19]. Descriptive statistics were used to characterize the sample: continuous variables are presented as mean ± standard deviation (SD) with 95% confidence intervals (CI), while categorical variables are reported as absolute frequencies (*n*) and percentages (%).

### Normality and bivariate analysis

The normality of continuous data distribution was assessed using the Kolmogorov–Smirnov test. Baseline comparisons between groups (CP vs. healthy controls) were conducted as follows:

- Categorical variables: Pearson’s Chi-square test was used to evaluate associations with clinical and respiratory indicators. Effect size was estimated using Cramer’s V (interpretable as small ≥ 0.1, medium ≥ 0.3, and large ≥ 0.5).
- Continuous variables: The independent Student’s *t*-test was employed to compare diaphragmatic ultrasound outcomes between groups. Homogeneity of variances was verified using Levene’s test. Effect sizes were quantified using Cohen’s *d*, interpreted as trivial (< 0.20), small (0.20–0.49), moderate (0.50–0.79), and large (≥ 0.80).

### Multivariate analysis

To assess independent associations between cerebral palsy and diaphragmatic outcomes, multiple linear regression models were fitted for each ultrasound variable, with group status (CP vs. control) as the primary predictor. All models were adjusted for age, sex, and weight, selected a priori based on biological plausibility. Medication use (dichotomized as use of antispastic or antiepileptic drugs), previous surgeries, and history of pulmonary disease were included as additional clinical confounders. History of pulmonary disease was defined as the presence of chronic or recurrent respiratory conditions documented in medical records or caregiver reports, including (i) bronchopulmonary dysplasia; (ii) recurrent pneumonia (≥ 2 physician-diagnosed episodes in the preceding 12 months); and (iii) chronic reactive airway disease or asthma requiring regular maintenance therapy. This composite variable was included as a covariate due to its potential impact on respiratory mechanics. No participant was receiving sedatives or neuromuscular blocking agents at the time of assessment.

For all regression models, the adjusted coefficient of determination (*R*^2^) and standardized beta coefficients were reported to indicate model fit and the strength of associations, respectively. Statistical significance was set at a two-tailed *p*-value < 0.05 for all inferential analyses.

## Results

### Study population and clinical characteristics

**Fig. 2:**
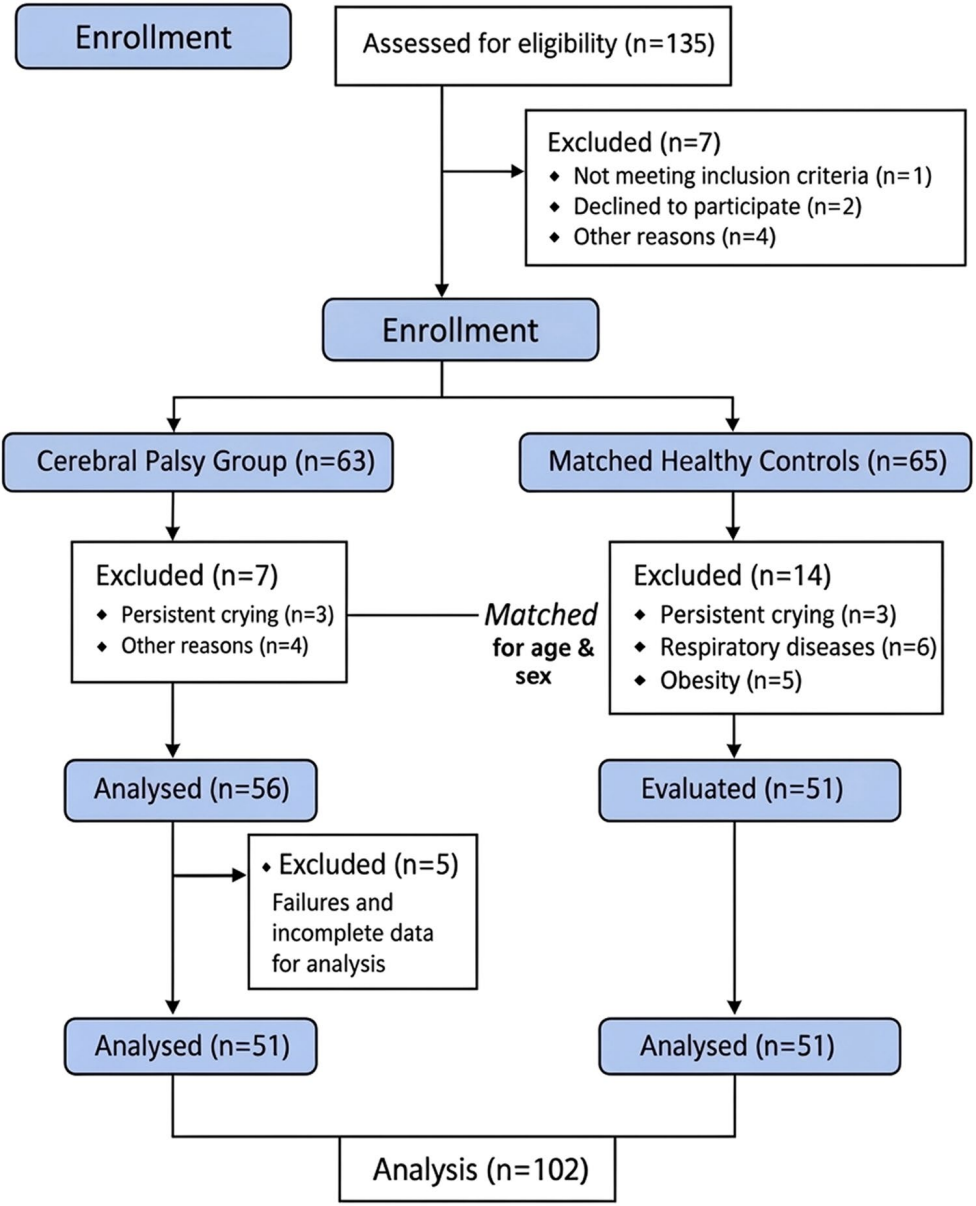
Flowchart of participant screening, eligibility, and inclusion, 2025

Following the screening and recruitment process detailed in the flow diagram (Fig. 2), a total of 102 participants were included in the final analysis (51 children with CP and 51 HC), with no missing data for primary outcomes. Matching was effective for age (*p* = 1.00) and height (*p* > 0.05). Although recruitment followed frequency-based matching for age and sex, post-screening eligibility resulted in a residual imbalance in sex distribution (*p* = 0.02); therefore, sex was included as a covariate in all multivariable models.

A significant anthropometric difference remained between groups, with children with CP presenting lower weight than controls (*p* = 0.00; Cohen’s *d* = 0.57). The CP cohort consisted exclusively of children with spastic hypertonia (100%), predominantly with bilateral motor involvement (67%) and a high proportion of severe functional impairment (55% classified as nonambulatory, GMFCS IV–V). Detailed demographic and clinical characteristics are provided in Table 1.

**Table 1:** Demographic characterization of the sample stratified by group, 2025

Variable	CP (*n* = 51)	HC (*n* = 51)	*p*-value
**Sex (female/male)**	21/30	32/19	0.02^*^
**Age (years, mean ± SD)**	7.1 ± 3.0	7.1 ± 3.0	1.00
**Height (kg, mean ± SD)**	20.7 ± 10.9	26.8 ± 10.7	0.00^*^
**Height (cm, mean ± SD)**	114.7 ± 17.8	122.1 ± 21.5	0.06
**Gross motor function (GMFCS), *n* (%)**			
Level I	4 (8%)	-	-
Level II	11 (22%)	-	-
Level III	8 (16%)	-	-
Level IV	7 (14%)	-	-
Level V	21 (41%)	-	-
**CP topographical type, n (%)**			
Spastic unilateral (hemiplegia)	17 (33%)	-	-
Spastic bilateral (total)	34 (67%)	-	-
Spastic diplegia (levels I–III)	13 (25%)	-	-
Spastic quadriplegia (levels IV–V)	21 (41%)	-	-
**Medications (%)**	71%	29%	0.01^*^
**Previous surgeries (%)**	22%	4%	0.00*
**Pulmonary disease (%)**	18%	0%	0.00*

*CP* cerebral palsy, *HC* healthy controls

### Diaphragmatic ultrasound profiles

Unadjusted comparative analysis revealed a distinct respiratory phenotype in the CP group (Table 2), that exhibited significantly greater inspiratory thickness compared to controls, higher contraction velocity, and markedly shorter inspiratory and expiratory times. The inspiratory excursion was similar to the health children group and expiratory excursion was significantly reduced in children with CP.

**Table 2:** Comparison of diaphragm muscle function between cerebral palsy and healthy children groups

Variable	CP (mean ± SD)	HC (mean ± SD)	*p*-value	CI 95%	Cohen’s *d* (effect size)
Inspiratory thickness (mm)	1.3 ± 0.4	1.2 ± 0.2	0.03*	− 0.24– − 0.01	0.4 (moderate)
Expiratory thickness (mm)	0.7 ± 0.3	0.7 ± 0.2	0.23	− 0.13– − 0.03	0.2 (small)
Inspiratory excursion (mm)	12.0 ± 3.7	13.3 ± 3.0	0.05	− 0.01– 2.63	0.4 (small)
Inspiratory time (s)	0.8 ± 0.3	1.0 ± 0.2	0.00^*^	0.10– 0.28	0.8 (large)
Contraction velocity (mm/s)	15.7 ± 5.3	13.6 ± 3.4	0.01^*^	− 3.95– − 0.42	0.5 (moderate)
Expiratory excursion (mm)	11.8 ± 3.6	14.0 ± 2.9	0.00^*^	0.92– 3.47	0.7 (moderate)
Expiratory time (s)	1.1 ± 0.4	1.3 ± 0.3	0.00^*^	0.09– 0.36	0.7 (moderate)
Relaxation velocity (mm/s)	10.5 ± 4.3	9.8 ± 2.7	0.30	− 2.16– 0.68	0.2 (small)

To determine whether the observed differences represented primary diaphragm dysfunction or were secondary to demographic, anthropometric, and clinical factors, after adjustment for age, sex, weight, medication use, prior surgeries, and history of pulmonary disease between CP and HC, most differences observed in the unadjusted analysis were no longer statistically significant.

Specifically, inspiratory and expiratory thickness, thickening fraction, inspiratory excursion, inspiratory time, contraction velocity, and relaxation velocity were not independently associated with CP (*p* > 0.05 for all).

In contrast, expiratory diaphragmatic excursion (*B* = − 2.02 mm; *p* = 0.03) and expiratory time (*B* = − 0.24 s; *p* = 0.01) remained significantly associated with CP after full adjustment, indicating a specific impairment in expiratory mechanics independent of confounding factors.

### Subgroup analysis by motor severity

Stratification by GMFCS level demonstrated a clear functional gradient. Nonambulatory children (GMFCS IV–V) showed more impaired diaphragmatic mechanics than ambulatory peers (GMFCS I–III), including reduced excursion in both respiratory phases (*p* < 0.01) and shorter inspiratory time (0.58 s *vs.* 0.74 s; *p* = 0.00). After adjustment, the between-group difference persisted primarily in the expiratory phase, consistent with the greater dependence of expiratory return on trunk stabilization and abdominal muscle coordination. Thickening fraction and velocity parameters did not differ across functional strata. Although absolute differences were modest, the selective reduction in expiratory excursion supports impaired expiratory control rather than diffuse diaphragmatic weakness.

## Discussion

This cross-sectional study compelling evidence that children and adolescents diagnosed with CP compared to their typically developing peers evaluated by diaphragmatic ultrasound, demonstrated that expiratory diaphragmatic excursion and expiratory time is associated with CP independent of age, sex, weight, medication use, prior surgeries, and history of pulmonary disease. Inspiratory and expiratory thickness, thickening fraction, inspiratory excursion, inspiratory time, contraction velocity, and relaxation velocity were not independently associated with CP. To the best of our knowledge, this is one of the few population-based studies to systematically apply diaphragmatic ultrasound (DU) to characterize respiratory muscle dysfunction in this vulnerable pediatric population. This finding reinforces earlier observations that respiratory impairment remains an underestimated but pivotal determinant of morbidity in CP [5, 8].

### The “pseudo-hypertrophy” phenomenon and anthropometric confounding

A primary and initially counterintuitive finding was the greater inspiratory thickness observed in children with CP. Although neuromuscular weakness and spasticity are expected to impair mechanical efficiency [20], multivariable analysis indicated that the apparent increase in thickness and contraction velocity was explained by lower weight rather than by a true morphometric advantage. After adjustment for anthropometric variables, these differences lost statistical significance. This suggests that previously reported alterations in diaphragmatic morphometry may partly reflect inadequate scaling for body size rather than intrinsic structural enlargement. Alternatively, the increased thickness may reflect chronic fiber shortening secondary to spasticity or thoracoabdominal distortion, producing a diaphragm that appears thicker but functions under suboptimal length-tension conditions [19, 21].

#### Expiratory dysfunction

After covariate adjustment, inspiratory and expiratory excursions showed distinct patterns. Although diaphragmatic excursion reflects net displacement, the two phases are not mechanically equivalent. De Troyer and Wilson (2015) [22] showed that inspiratory descent is primarily driven by active diaphragmatic contraction, increasing thoracic volume and promoting air intake. In this phase, the abdominal muscles relax, allowing the abdomen to expand and facilitating the downward movement of the diaphragm. In addition, the increase in intra-abdominal pressure helps to stabilize and enhance the action of the diaphragm on the lower ribs. During passive expiration, the diaphragm relaxes and returns to its resting position due to pulmonary elastic recoil.

In contrast, the expiratory return of the diaphragm relies heavily on coordinated abdominal recruitment, trunk stabilization, intra-abdominal pressure generation, and efficient thoracoabdominal coupling, mechanisms commonly compromised in CP [23]. This biomechanical dependence plausibly explains why expiratory excursion remained reduced in the CP group despite rigorous adjustment for confounders.

Although the absolute differences between groups were small, the persistence of reduced expiratory excursion and shorter expiratory time after full adjustment suggests passive expiratory-phase mechanical deficit. The expiratory return of the diaphragm requires coordinated abdominal recruitment, trunk stabilization, intra-abdominal pressure generation, and thoracoabdominal coupling, mechanisms often compromised in CP. While these measures do not directly quantify cough performance, they may represent an underlying neuromechanical vulnerability that could contribute to reduced airway clearance capacity. These findings should therefore be interpreted as hypothesis-generating regarding potential mechanisms of respiratory morbidity in this population.

#### Neuromechanical inefficiency

The kinematic profile observed, characterized by higher contraction velocity despite reduced excursion and shortened inspiratory time, likely reflects a constrained, inefficient breathing pattern rather than enhanced performance. From a neuromechanical standpoint, this suggests a mismatch between neural drive (velocity) and mechanical output (displacement). The diaphragm contracts rapidly but encounters early mechanical braking due to chest wall stiffness or poor thoracoabdominal coordination, resulting in a “rapid-shallow” pattern that fails to generate adequate tidal volume [19, 21].

#### Clinical implications and severity

Our data highlight that children with severe motor impairment (GMFCS levels IV–V) exhibit the most profound diaphragmatic restrictions. This aligns with large-cohort evidence linking gross motor dysfunction to respiratory morbidity [24, 25]. Such findings reinforce the urgent need to incorporate respiratory muscle training (RMT) and chest wall mobility strategies into conventional neurorehabilitation [12]. However, our results also serve as a cautionary note: clinical interpretation of DU must be adjusted for weight. Without this correction, clinicians risk misinterpreting a “thick” diaphragm as functionally preserved, potentially overlooking the latent expiratory dysfunction that poses the true risk for respiratory failure.

#### Strengths and limitations

A major strength of this study is the use of diaphragm ultrasonography, a child-friendly, reproducible, and noninvasive technique that is particularly valuable in populations for whom invasive respiratory assessments are neither feasible nor ethical. The standardized acquisition protocol and excellent intra-rater reliability further enhance the robustness of the measurements.

This study also presents limitations. The cross-sectional design does not allow conclusions regarding temporal changes or the progression of diaphragmatic mechanics. Another important limitation relates to stature assessment. Because standing height cannot be reliably obtained in many children with CP, particularly those with contractures, scoliosis, or limited postural control (different measurement approaches were required across participants. Standing stadiometry was used when feasible, whereas knee-height) derived equations were applied in nonambulatory children. Although these methods are validated, the resulting heterogeneity may introduce measurement variability. This methodological constraint, combined with the observed multicollinearity between height, age, and weight, justified the exclusion of height from multivariable models and the selection of weight as a more stable and physiologically relevant anthropometric covariate.

Despite matching for age and sex, skeletal deformities such as scoliosis were not quantified radiologically. Although key covariates were included in the adjusted analyses, residual confounding from unmeasured musculoskeletal factors cannot be completely excluded. Future longitudinal studies incorporating spinal imaging and repeated DU assessments may clarify the impact of these structural influences on respiratory mechanics in children with CP.

## Conclusion

Children with cerebral palsy exhibited reduced expiratory diaphragmatic excursion and shorter expiratory time compared to typically developing peers, independently of age, sex, weight, medication use, prior surgeries, and pulmonary history. In contrast, inspiratory parameters were not independently associated with cerebral palsy. These findings identify expiratory dysfunction as a key physiological feature in this population, supporting a central role for impaired abdominal muscle coordination in diaphragmatic performance. Diaphragm ultrasonography emerges as a promising noninvasive tool for functional assessment, with potential implications for risk stratification and targeted respiratory interventions.

## Supplementary Information

Below is the link to the electronic supplementary material.

ESM 1 (DOCX 31.8 KB)

## Data Availability

The datasets generated and analyzed during the current study are not publicly available due to restrictions related to patient confidentiality but are available from the corresponding author on reasonable request.

## Abbreviations

CAPES: Coordenação de Aperfeiçoamento de Pessoal de Nível Superior
CAAE: Certificate of Presentation of Ethical Review
CISAM/UPE: Centro Integrado de Saúde Amaury de Medeiros / Universidade de Pernambuco
CP: Cerebral palsy
DE: Diaphragmatic excursion
DU: Diaphragm ultrasound
GMFCS: Gross Motor Function Classification System
HC: Healthyc
IBM SPSS: International Business Machines/Statistical Package for the Social Sciences
LAFIC: Cardiopulmonary Physiotherapy Laboratory
M-mode: Motion mode
RMT: Respiratory muscle training
SD: Standard deviation
STROBE: Strengthening the Reporting of Observational Studies in Epidemiology
UPE: Universidade de Pernambuco
UFPE: Universidade Federal de Pernambuco
US: Ultrasonography
